# Alveolar macrophages: Achilles’ heel of SARS-CoV-2 infection

**DOI:** 10.1038/s41392-022-01106-8

**Published:** 2022-07-19

**Authors:** Zhenfeng Wang, Shunshun Li, Bo Huang

**Affiliations:** 1 Department of Immunology & National Key Laboratory of Medical Molecular Biology, Institute of Basic Medical Sciences, Chinese Academy of Medical Sciences (CAMS) & Peking Union Medical College, 100005 Beijing, China; 2 Department of Immunology, Basic Medicine College, China Medical University, 110122 Shenyang, Liaoning China; 3 Department of Biochemistry & Molecular Biology, Tongji Medical College, Huazhong University of Science & Technology, 430030 Wuhan, China

**Keywords:** Infection, Innate immunity, Innate immune cells

## Abstract

The coronavirus disease 2019 (COVID-19) pandemic has caused more than 6.3 million deaths to date. Despite great efforts to curb the spread of severe acute respiratory syndrome coronavirus 2 (SARS-CoV-2), vaccines and neutralizing antibodies are in the gloom due to persistent viral mutations and antiviral compounds face challenges of specificity and safety. In addition, vaccines are unable to treat already-infected individuals, and antiviral drugs cannot be used prophylactically. Therefore, exploration of unconventional strategies to curb the current pandemic is highly urgent. Alveolar macrophages (AMs) residing on the surface of alveoli are the first immune cells that dispose of alveoli-invading viruses. Our findings demonstrate that M1 AMs have an acidic endosomal pH, thus favoring SARS-CoV-2 to leave endosomes and release into the cytosol where the virus initiates replication; in contrast, M2 AMs have an increased endosomal pH, which dampens the viral escape and facilitates delivery of the virus for lysosomal degradation. In this review, we propose that AMs are the Achilles’ heel of SARS-CoV-2 infection and that modulation of the endosomal pH of AMs has the potential to eliminate invaded SARS-CoV-2; the same strategy might also be suitable for other lethal respiratory viruses.

## Introduction

Respiratory viruses are the most frequent pathogens causing human diseases, with a significant impact on morbidity and mortality worldwide.^1^ In addition to influenza virus, respiratory syncytial virus, parainfluenza virus, rhinovirus and adenovirus, coronaviruses are also common circulating respiratory viruses that induce symptoms of cough, fever, sore throat, and headache.^2–5^ Unlike conventional coronavirus subtypes, the currently spreading severe acute respiratory syndrome coronavirus 2 (SARS-CoV-2) can be fatal, especially the Delta variant.^6^ Avian flu viruses (H5N1 and H7N9) and Middle East respiratory syndrome coronavirus (MERS-CoV) are also fatal.^7,8^ Thus, questions about why some respiratory viruses are mild but some lethal and how deadly respiratory viruses cause death in infected people have been raised. The primary function of the lung is to exchange oxygen (O_2_) and carbon dioxide (CO_2_), which take place at alveoli. We argue that deadly respiratory viruses invade alveoli and disrupt O_2_ and CO_2_ exchange, leading to death. In this review, we focus on the earliest immune events after SARS-CoV-2 invade the alveoli and propose that the polarizing state of alveolar macrophages (AMs) is the major determining factor for the outcome of viral infection.

### Entry of SARS-CoV-2 into cells

**Fig. 1:**
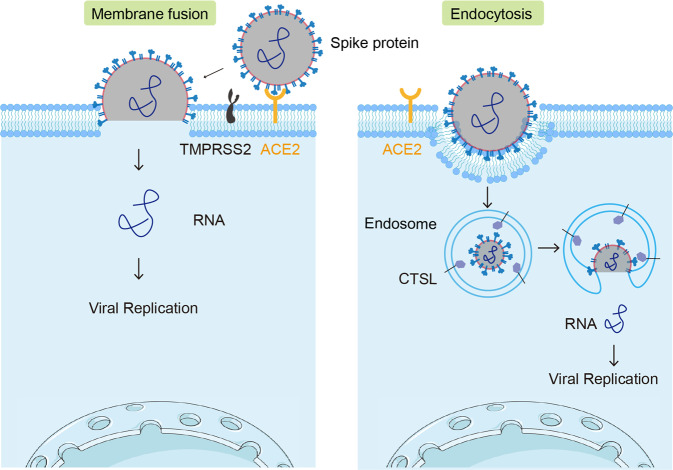
SARS-CoV-2 invades cells via two distinct pathways. Viral entry can be mediated by membrane fusion or endocytosis. The former involves binding of the SARS-CoV-2 S protein to the ACE2 receptor enriched in host cells and cleavage by the cell membrane enzymes furin and TMPRSS2. S protein cleavage exposes hydrophobic amino acid residues, triggering fusion of the proximal viral and plasma membranes, and the viral RNA is released into the cytoplasm. In the endocytosis route, viral particles are enclosed in the endosome following uptake. With gradual acidification of the endosomes by v-ATPase recruitment, CSTL activity is recovered to cleave the S protein, leading to fusion of the viral and endosomal membranes and subsequent release of the viral RNA into the cytoplasm. In host cells expressing low levels of TMPRSS2 or macrophages, the endocytosis pathway of SARS-CoV-2 uptake may be used

SARS-CoV-2 is a positive-sense single-stranded RNA virus, comprising a viral RNA-containing inner nucleocapsid and a spike (S) protein-containing outer membrane.^9,10^ To infect cells, the viral S protein must be cleaved, allowing entry of the viral genomic RNA into the cytoplasm.^11,12^ Viral entry may be mediated by two undressing means. The first is via binding of the S protein to the ACE2 receptor on host cells such as alveolar epithelial cells, followed by the S protein cleavage by the membrane enzymes furin and TMPRSS2.^11,12^ Such hydrolyses lead to exposure of the hydrophobic amino acid residues of the S protein, which insert into the plasma membrane and trigger interaction between proximal viral and plasma membranes.^13,14^ As a consequence, the apertures in viral and plasma membranes are formed, which is called membrane fusion, allowing for the release of the viral RNA into the cytoplasm (Fig. 1). The second pathway starts with endocytosis. Following uptake, the viral particle is enclosed in endosomes or phagosomes.^15,16^ With gradual acidification, the activity of the endosomal enzyme cathepsin L (CTSL) is recovered, and it begins to cleave the S protein, leading to the fusion of viral and endosomal membranes and subsequent release of viral RNA into the cytoplasm (Fig. 1).^11,17^ Once SAS-CoV-2 genomic RNA enters the cytoplasm, the host machinery is used to complete viral replication, assembly, and viral particle release.^18^ The newly synthesized virions are then able to infect other target cells for viral spread.

### Other receptors for SARS-CoV-2 entry

Soon after the COVID-19 pandemic began, ACE2 was identified as the major receptor for SARS-CoV-2,^19,20^ which was similar to severe acute respiratory syndrome coronavirus (SARS-CoV), which emerged in 2003. Nevertheless, several studies have shown that cells in certain infected tissues or organs display low expression of ACE2, suggesting that other receptors or molecules are involved in the SARS-CoV-2 entry process.^21–23^ Indeed, AXL (tyrosine-protein kinase receptor UFO),^24^ KREMEN1 (Kringle-containing protein marking the eye and the nose protein 1),^25^ ASGR1 (asialoglycoprotein receptor 1),^25^ and CD147^22,26^ have been shown to be involved in SARS-CoV-2 entry independent of ACE2. These molecules provide SARS-CoV-2 with distinct pathways for entry into different host cells. In addition, HS (heparan sulfate),^20^ sialic acid,^27^ lectin receptors,^23^ NRP1 (neuropilin 1),^12,28^ and SR-B1 (scavenger receptor B type 1)^29^ have been reported as coreceptors for SARS-CoV-2. Considering that the S protein is extensively decorated by glycans,^30^ it is not surprising that lectin receptors can promote viral infection through the nonspecific attachment to the glycan shield of SARS-CoV-2.^31^ For instance, the NTD of SARS-CoV-2 S1 subunit has a sialic acid-binding pocket that can interact with various sialoproteins or glycoproteins.^32,33^ Consistently, although ACE2 is used to adsorb SARS-CoV-2, tumor cell-derived microparticles (T-MPs) retain approximately 10% capacity to adsorb viral particles under ACE2-deficient conditions.^16^

### Epithelial cells of bronchus, bronchiole, and alveoli

The respiratory tract is composed of two parts including upper and lower respiratory tracts. The nasal cavity, pharynx, and larynx form the upper respiratory tract; the trachea, bronchi, bronchioles, and alveoli form the lower respiratory tract.^34^ The Trachea and bronchi are covered by different types of epithelial cells.^35^ Among them, ciliated columnar cells account for the majority; the rest are goblet, basal and neuroendocrine cells.^36^ Goblet cells secrete mucus, which includes IgA, lysozymes, lactoferrin, and peroxidases.^37,38^ When air is inhaled, not only oxygen but also air particles including viral particles are inevitably taken up into the respiratory tract, where the mucus can adsorb particles from 2 to 10 μm,^39^ and ciliated columnar cells wobble the cilia to move the mucus.^40^ As a result, sputum is formed and excreted via coughing.^37,40^ Regardless of this protective mechanism, some xenobiotics are still able to enter the end of the respiratory tract, where numerous balloon-like sac structures called alveoli are formed.

**Fig. 2:**
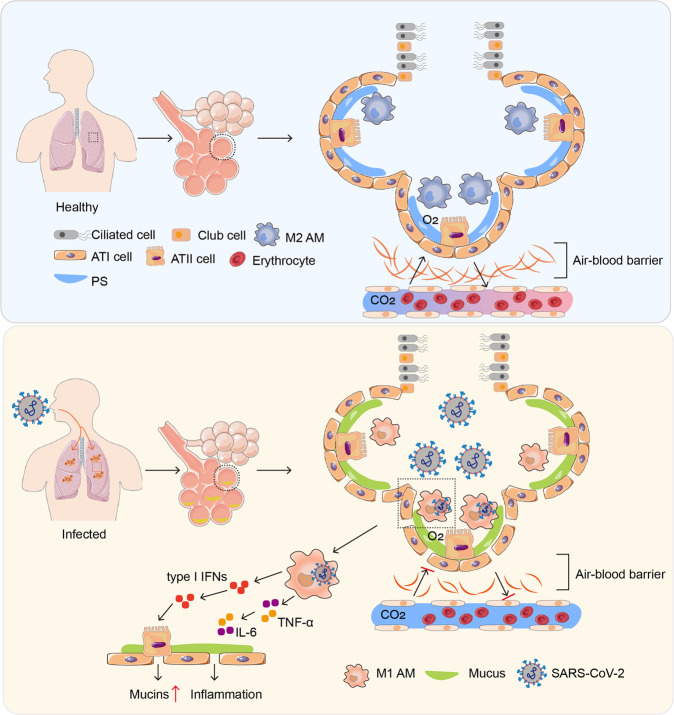
The pathological features of the lung in SARS-CoV-2-infected patients. In healthy conditions, alveolar epithelia are composed of alveolar epithelial type I (ATI) and ATII cells. ATI cells are squamous cells covering ~95% of the alveolar internal surface area; the other 5% are covered with cuboidal ATII cells, which secrete pulmonary surfactant. Alveolar macrophages (AMs) reside in airspaces, accounting for ~95% of alveolar immune cells, and safeguard against most inhaled irritants. The lung air–blood barrier acts as the site for O_2_ and CO_2_ exchange. Nevertheless, a certain amount of viruses can cause AMs polarization toward the M1 phenotype. Proinflammatory cytokines secreted by SARS-CoV-2-infected M1 AMs abrogate PS production by ATII cells, allowing the new virions released from M1 AMs to gain access to ATII cells. The type I interferons released by infected M1 AMs stimulate ATII cells to produce mucus. However, accumulation of alveolar mucus affects the blood-gas barrier, leading to impaired O_2_–CO_2_ exchange

Alveoli vary in size and shape, with an average diameter of 200 μm. An adult has 300 million to 400 million alveolar structures in each lung, with a total area of 140 m^2^.^41,42^ The alveolar epithelium is composed of alveolar epithelial type I (ATI) and type II (ATII) cells. ATI cells are squamous and cover ~95% of the alveoli surface area^43^ and participate in the exchange of oxygen and carbon dioxide as the lung air–blood barrier.^44^ ATII cells are cuboidal and cover ~5% of the alveoli surface area. ATII cells synthesize, secrete, and recycle all components of the surfactant that regulates alveolar surface tension in the lungs (Fig. 2).^43^

### Alveolar surfactant structure and function

The alveolar surface is initially covered by a very thin aqueous film termed the hypophase. The hypophase is delimited at the alveolar face by the surfactant lining layer and at the septal face by the alveolar epithelium.^45^ This water film is formed by a variety of proteins known as aquaporins in the cell membrane of type I alveolar cells, which facilitate intracellular water molecule transport across the plasma membrane.^46,47^ Thus, the hypophase can be considered a reaction milieu for extracellular biochemical processes as well as a kind of medium for intra-alveolar cells such as AMs. To keep alveoli open, dry, and clean, the hypophase is further overlain by a film of pulmonary surfactant (PS),^48^ which is a complex and active material consisting of proteins and lipids.^49,50^ PS is composed of 80–90% lipids by weight and ~10% proteins.^49,51,52^ Because air is more hydrophobic than water, the fatty acid tails of the surfactant lipids touch the air to form a film.^53^ More than 80% of the lipids are phospholipid (PL); 5–10% are neutral lipids, mainly cholesterol. The major PL components are saturated phosphatidylcholine (PC), phosphatidylglycerol (PG), phosphatidylethanolamine (PE), and phosphatidylinositol (PI).^51,54^ In addition to the surfactant lipids, PS contains four specific proteins: SP-A, SP-B, SP-C, and SP-D.^53^ The larger hydrophilic glycoproteins SP-A (32 kDa) and SP-D (43 kDa) are water soluble and mainly involved in innate host defense. SP-B (8.7 kDa) and SP-C (4.2 kDa) are relatively smaller hydrophobic proteins embedded in and between the phospholipid layers.^53,54^ SP-A is the most abundant protein, accounting for ~5% of PS by weight.^53^ Interaction between the lipids and SPs may help to maintain a stable surfactant film stable during breathing cycles.^51^ In general, PS complexes are assembled in ATII cells. The lipid fraction and surfactant proteins are stored tightly in lamellar bodies (LBs), a spherical structure with a diameter of 1–3 μm.^55,56^ When the LB-limiting membrane fuses with the plasma membrane of the cells, LBs are secreted through the fusion pore, triggered by the surface tension, hydration, pH, and calcium concentration.^55,57^ PS reduces the surface tension in the lung surface liquid and prevents alveolar collapse during expiration by decreasing elastic recoil.^53,56^

### Origin, functions, and plasticity of alveolar macrophages

**Fig. 3:**
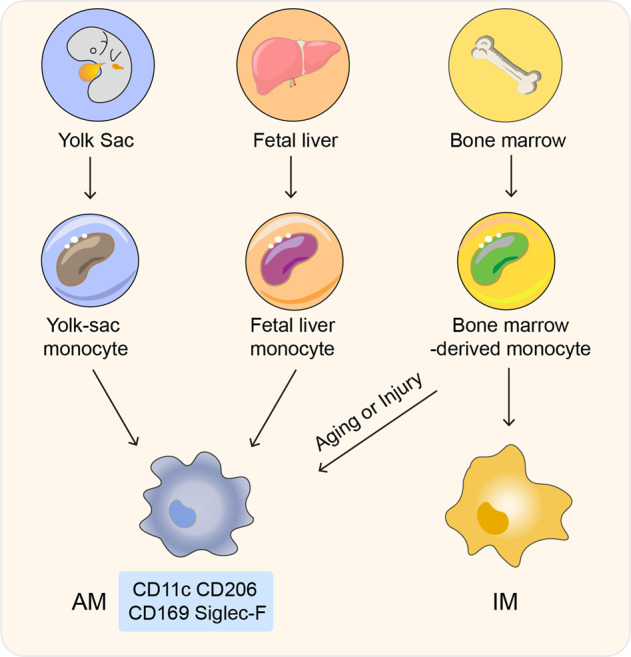
The origin of lung macrophages in mice. Lung macrophages mainly include two populations, AMs and interstitial macrophages (IMs). IMs originate from bone marrow-derived monocytes and are maintained by circulating monocytes. Unlike IMs, AMs are established prior to birth in the yolk sac, where erythromyeloid progenitors (EMP) develop at embryonic day (E) 8.5. Subsequently, progenitors migrate to the fetal liver, generating monocyte clones at E12.5. After birth, bone marrow-derived monocytes can be recruited to alveoli in aging mice or injured mice, as a supplement for AMs. In fact, in the steady-state, AMs maintain themselves by self-renewal. Murine AMs highly express CD11c, CD206, CD169, and Siglec-F

**Fig. 4:**
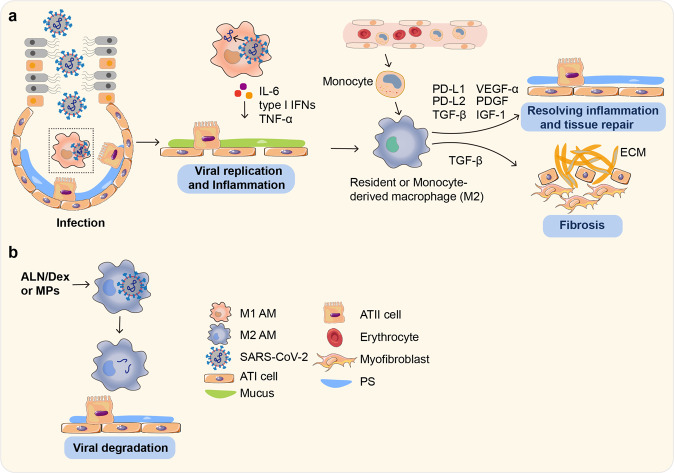
M1-like and M2-like AMs involve in SARS-CoV-2 infection differently. **a** M1-like AMs have more acidic endosomes than M2-like AMs, leading to release of the viral RNA from endosomes into the cytoplasm for replication. Meanwhile, proinflammatory cytokines, including TNF-α and IL-6, are upregulated in virus-infected M1 AMs, causing lung injury. Bone marrow-derived monocytes migrate into alveoli to replenish AMs during SARS-CoV-2 infection. These cells together with resident AMs may contribute to inflammation. However, they can also be polarized into potential M2-like phenotype by IL-4 and IL-13 stimulation, producing a variety of factors that facilitate tissue repair and resolving inflammation. If the process loses its balance, undesirable repair processes can cause tissue fibrosis. **b** Microparticles (MPs), Alendronate (ALN), and Dexamethasone (Dex) can promote viral degradation by polarizing AMs into M2 phenotype. M2-like AMs have a higher endosomal pH, which hinders CTSL activity and subsequent viral RNA release, leading to the delivery of viruses to lysosomes for degradation

In addition to maintaining the structure of alveoli, PS acts as a separator, which blocks access of inhaled particles or pathogens to alveolar epithelial cells.^58^ These xenobiotics, however, can gain access to and are taken up by AMs, which reside in the PS surface and the underlying hypophase and account for ~95% of resident alveolar immune cells.^15,59^ Lung macrophages mainly include two populations, AMs and interstitial macrophages (IMs).^60,61^ Unlike IMs, which originate from adult hematopoiesis and are maintained by circulating monocytes,^62–64^ AMs are established in the yolk sac, where erythromyeloid progenitors (EMP) migrate to the fetal liver to form fetal progenitors that give rise to mature AMs in a steady and local self-renewal state.^64–66^ In response to local inflammatory stimuli, blood monocyte progenitors may migrate to the lung and yield AMs (Fig. 3).^65^ As alveolar guardians, AMs physiologically display an M2 phenotype,^67,68^ expressing the cell-surface protein mannose receptor C type 1 (MRC1, CD206), which facilitates endocytosis and phagocytosis.^15^ Thus, AMs act as scavengers to continually phagocytose inhaled xenobiotics to maintain lung homeostasis. However, because the respiratory tract is an open system, AMs are also endowed with high plasticity, allowing AMs to switch from an anti-inflammatory M2 state to a proinflammatory M1 state in response to pathogenic invasion.^69^ By recognizing pathogen-associated molecular patterns (PAMPs) with pattern recognition receptors (PRRs) such as Toll-like receptors and NOD-like receptors, AMs can rapidly mobilize NF-κB and MAPK signaling to trigger inflammatory responses.^70–72^ Thus, AMs are the true first-line defender against viral or bacterial invasion of alveoli. Furthermore, the polarization state may profoundly influence the ability of AMs to control SARS-CoV-2 infection. Our findings provide evidence that M1 AMs have more acidic endosomes but more alkaline lysosomes than M2 AMs. These differences cause SARS-CoV-2 to mostly replicate in M1 AMs, but not in M2 AMs which are responsible for early viral control (Fig. 4).^15^

### M2-polarized AMs limit SARS-CoV-2 infection

Dry cough is a typical symptom of SARS-CoV-2 infection,^73,74^ indicating that the virus mainly invades the alveoli. Given the separation of PS, viral particles are prevented from gaining access to alveolar epithelial cells, which, however, are easily detected and taken up by AMs.^75^ As mentioned above, AMs are likely to exhibit an M2-like phenotype under normal conditions. In a recent study, we found that M2 AMs have a higher endosomal pH but a lower lysosomal pH.^15^ This increased pH inhibits the enzymatic activity of CTSL and impedes the release of viral RNA into the cytoplasm; in contrast, the virus is delivered from endosomes to lysosomes, where it is degraded by acidic lysosomal enzymes.^15^ Thus, M2 AMs possess the ability to degrade SARS-CoV-2 and limit viral infection and spread, which might explain the high percentage of asymptomatic SARS-CoV-2-infected people. Indeed, M2 macrophages exhibit an anti-inflammatory cytokine profile that favors tissue repair.^76–78^ When tissues are injured or infected, macrophages differentiate toward the anti-inflammatory phenotype and become the dominant population, which is characterized by low production of IL-12 but high levels of IL-10 and TGF-β.^79,80^ Moreover, the differentiated macrophages express PD-L1, PD-L2, and other immunosuppressive receptors to quench inflammation and accelerate the repair process (Fig. 4a).^80,81^ Several studies have reported that anti-inflammatory and tissue-repair-related genes, such as *Retnla* and *Chil3*, are upregulated in IL-4 stimulated macrophages.^82,83^

In addition to a direct effect on SARS-CoV-2 infection, M2 macrophages may have an indirect effect on viral pathogenesis. M2 cells promote blood vessel development and cellular proliferation by producing growth factors such as platelet-derived growth factor (PDGF), vascular endothelial growth factor (VEGF)-α, and insulin-like factor 1 (IGF-1). Therefore, M2 macrophages play a vital role in tissue repair after injury.^80^ Although SARS-CoV-2 causes a mild respiratory infection, ~5% of patients develop ARDS (Fig. 4a). There are three phases of the pathophysiology that describes lung injury, as follows: (1) the exudative phase is the first step in which innate immune cells cause damage to the alveolar endothelium and result in accumulation of protein-rich edema fluid in alveoli; (2) the proliferative phase is characterized by a repair process by which the alveolar integrity is rebuilt and edema is reabsorbed; and (3) the fibrotic phase, the final process, occurs only in a subset of patients and is highlighted by respiratory failure and a high mortality rate.^84^ Clinical observation shows that accumulate excessive extracellular matrix (ECM) accumulates in SARS-CoV-2-infected patients with severe disease who develop ARDS, leading to fibrosis development, alveolar destruction, and lung dysfunction.^85,86^ Bone marrow-derived monocytes can migrate to alveoli to replenish AMs when the lung is injured.^63^ Indeed, analysis of gene sets for COVID-19 has shown that monocyte-derived macrophages are highly similar to idiopathic pulmonary fibrosis (IPF)-associated macrophages and that several fibrosis-associated genes are enriched in monocyte-derived macrophages which are similar to the pulmonary CD163^+^ macrophages accumulating in COVID-19.^86^ CD163 is also considered a specific marker for M2 macrophages.^87^ On the other hand, knockout of the anti-apoptotic protein C-FLIP in monocytes reduces infiltration of monocyte-derived macrophages in lung tissue and alleviates pulmonary fibrosis in mice.^88^ These findings together suggest that monocyte-derived AMs rather than lung-settled AMs are involved in the formation of pulmonary fibrosis. In conclusion, early control of inflammation and viral replication is key for inhibiting lung fibrosis in SARS-CoV-2- infected patients.

### M1-polarized AMs accelerate SARS-CoV-2 infection

Under the physiological conditions, AMs usually exist in an M2 phenotype;^15^ however, AMs with an M1 phenotype may also be present in a small population of people due to systemic or local causes. Relative to M2 counterparts, M1 AMs have more acidic endosomes.^15^ Thus, the S protein of phagocytosed SARS-CoV-2 is easily cleaved by activated CTSL,^13,89^ leading to viral and endosomal membrane fusion and viral RNA release into the cytoplasm. Considering the anti-inflammatory nature of M2 AMs, it might be assumed that these cells are stronger than M1 in taking up exogenous materials.^90,91^ However, our findings revealed that M1 AMs have a stronger phagocytic ability because they are softer than M2 AMs, thus allowing M1 AMs to more easily deform and take up SARS-CoV-2 particles,^15^ facilitating viral spread. Moreover, such increased phagocytosis might promote M1 AMs to take up more lipid components of the PS.^92^ At the same time, the proinflammatory cytokines released by M1 AMs might disturb ATII cells to secrete surfactant components,^93^ thus disrupting the normal structure of PS and leading to access of viral particles to ATII cells. In addition, although they have more acidic endosomes, M1 AMs actually possess more alkaline lysosomes than M2 AMs,^15,94^ and such a high lysosomal pH impairs degradation of the virus (Fig. 4a). M2 AMs limit SARS-CoV-2 spread, but viral RNA can act as a PAMP to induce the release of proinflammatory cytokines.^95,96^ These cytokines, including TNF-α, may favor AMs polarization toward an M1-like phenotype.^97^ Hence, if the viral load reaches a certain level in alveoli, SARS-CoV-2 might reset M2 AMs toward the M1 phenotype, facilitating viral spread.

### M1-polarized AMs disturb O_2_–CO_2_ exchange

The basic function of the lung is O_2_ and CO_2_ exchange, which occurs at alveolar sites.^44,98–100^ O_2_ is inhaled from air, and CO_2_ is generated by tissue cells through the tricarboxylic acid (TCA) cycle.^101,102^ As a waste product, CO_2_ is released from cells into the interstitial fluid and further diffuses into capillary vessels. Subsequently, CO_2_ is brought to the alveoli and expelled by crossing the blood-gas barrier during exhalation; O_2_ is brought from alveoli into the alveolar-capillary blood for exchange. Normally, O_2_ and CO_2_ exchange between alveoli and pulmonary capillary blood is achieved through a passive diffusion process.^103^ However, alteration of the thickness of the blood-gas barrier can influence O_2_ and CO_2_ exchange, and one such factor is mucus. Autopsy has revealed copious amounts of gray-white viscous fluid in the lungs of SARS-CoV-2-infected patients and single-cell RNA-seq analysis has revealed mucin expression in lung epithelial cells.^104,105^ Considering that alveoli are dry and clean, how is this mucus induced? In response to SARS-CoV-2 infection, ATII cells produce mucins. Exploring the molecular basis shows that both IFN-β and IFN-γ stimulate ATII cells to produce mucins through the IDO-kynurenine-AhR pathway.^103^ This mucin production induced by IFNs might be an evolutionary response to protect alveolar epithelial cells, which unfortunately is usurped by SARS-CoV-2 to cause pathological lung damage. Although SARS-CoV-2 does not directly induce mucin production in ATII cells, the virus does stimulate M1 AMs to produce both IFN-β and IFN-γ,^106^ thus indirectly inducing mucins. In addition to mucin production, M1 AM-released proinflammatory cytokines can increase capillary permeability, inducing capillary leakage and attracting a large number of innate immune cells into the alveolar space to further amplify the inflammatory reaction.^106–109^ As a consequence, accumulated mucus and exudate may impede O_2_ and CO_2_ exchange and cause lung pathological damage.

### SARS-CoV-2 variants break through the M2 limitation for the ultrafast spread

Epidemiological evidence has demonstrated that current vaccines have reduced effectiveness against SARS-CoV-2 Delta or Omicron variant than wild-type SARS-CoV-2, though they do have the effect on reducing severe disease and mortality.^110–112^ This indicates that the vaccine-generated anti-spike antibody has the protective effect, which, however, cannot exert the effect at the earliest phase during the infection. This may be explained by the PS which prevents antibody molecules from gaining access to the inhaled SARS-CoV-2 variant. Such variant, however, can be taken up by physiological M2 AMs, which must break through the endosomal limitation of M2 AMs. Cleavage of viral S protein by endosomal CTSL relies on an acidic pH.^13^ However, mutated S protein does not seem to alter this. On the other hand, considering that a low pH facilitates the protonation of substrate molecules spike protein protonation is promoted with the Delta variant, which harbors the T478K, P681R, and L452R mutations, due to the increase in lysine (K) or and arginine (R) amino groups.^13,113^ Thus, the Delta variant is likely to show enhanced endosomal spike protein cleavage by CTSL via enhanced protonation. Indeed, we found that CTSL is able to cleave the Delta S protein rather than that of Beta or Gamma at pH 6.5.^13^ Even under pH 6.0 condition, cleavage of the Beta or Gamma S protein by CTSL is weak. Thus, the general presence of M2 AMs in normal human alveoli and the relatively basic endosomal pH may explain why many SARS-CoV-2-infected people have no clinical symptoms or have only mild symptoms. Unfortunately, the Delta variant carries basic amino acid mutations, which break the limitation of a more alkaline endosomal pH and lead to viral replication in M2 AMs, achieving an ultrafast spread in populations.^13^ Similar to the Delta variant, the current dominant Omicron variant harbors many basic amino acid mutations (G339D, S371L, S373P, S375F, K417N, N440K, G446S, S477N, T478K, E484A, Q493R, G496S, Q498R, N501Y, Y505H, T547K, D614G, H655Y, N679K, P681H).^114^ Thus, it is easy to deduce ultrafast spread with Omicron via mutated basic amino acids.

### AMs are a potential target to curb the pandemic

Vaccines and antiviral drugs are two primary means to greatly prevent viral spread in the current pandemic.^16^ However, vaccines are unable to treat already-infected patients and may become invalid due to viral mutations.^115,116^ On the other hand, antiviral drugs cannot be used as prophylactic agents, and screening small compound(s) to target the viral life cycle is nonspecific and unsafe.^16^ Therefore, exploration of unconventional strategies to curb the spread of SARS-CoV-2 is highly desirable and urgently needed. The majority of people have no symptoms or have only mild symptoms following SARS-CoV-2 infection, which suggests that the human immune system can clear SARS-CoV-2 infection at a very early stage. This information and the above analyses all converge on AMs as the key factor to curb SARS-CoV-2 infection at the very early stage. Cells have the ability to produce extracellular vesicles, including exosomes and microparticles (MPs, also known as microvesicles or ectosomes).^117–119^ Unlike exosomes, which are smaller in size (30–100 nm) and released from endosomes,^117,118^ MPs, with sizes of 100–1000 nm, are released from the plasma membrane by many cell types in response to stimuli.^119^ As nonsynthetic nanocarriers, MPs have been used to deliver chemotherapeutic drugs for cancer treatment.^120–123^ Of note, extracellular vesicles containing ACE2 can be used as decoys to capture SARS-CoV-2.^124,125^ In addition, synthetic decoy nanoparticles and nanosponges can be used to trap SARS-CoV-2 as well as to adsorb proinflammatory cytokines with antibody conjugation.^125^ Unlike these approaches, we used ACE2-overexpressing A549 tumor cells to prepare ACE2-MPs, which have the following merits: (1) high efficiency of adsorbing viral particles; (2) ability to elevate the endosomal pH but to decrease the lysosomal pH of macrophages; and (3) resetting macrophages from a proinflammatory M1 to an anti-inflammatory M2 phenotype.^16^ Through airway administration, inhaled ACE2-MPs adsorb SARS-CoV-2 at alveolar sites and are taken up by AMs. However, MPs cause a decrease in the H^+^ concentration in the endosomal lumen, increasing the endosomal pH. This is because the cholesterol in ACE2-MPs is the oxidized form (25-hydroxy cholesterol), which integrates into the lipid raft structure of the endosomal membrane, interfering with vacuolar H^+^-ATPase, enforcing a limitation on SARS-CoV-2 release, and delivering the virus to lysosomes for degradation.^16^ In addition to the effect on pH, ACE2-MPs regulate AM-induced inflammation. It has been reported that T-MPs polarize macrophages toward the M2 phenotype.^126^ Although SARS-CoV-2 stimulates AMs to upregulate the expression of proinflammatory cytokines and antiviral type I interferons, ACE2-MP treatment can inhibit the induction of proinflammatory cytokines but not type I interferons, suggesting that the inflammatory and antiviral responses of AMs can be separated by ACE2-MPs.^16^ Our previous studies also demonstrated that T-MPs also activated the cGAS/STING signaling pathway in both DCs and macrophages.^126,127^ However, in macrophages, cGAS/STING activates TBK1, which in turn activates the signaling molecules STAT3 and STAT6, rather than conventional IRFs. Phosphorylation of STAT6 induces expression of arginase 1,^126^ a typical marker for M2 AMs.^69^ Although type I interferons can be regulated by the cGAS/STING pathway,^128,129^ whether and how MP treatment regulates the expression of IFNβ in the virus-infected AMs remains to be further investigated. Taken together, cellular MPs as advanced materials may provide potential means to curb SARS-CoV-2 by targeting AMs.

Apart from MPs, screening old drugs brings new hope to curb SARS-CoV-2 by targeting AMs. Bisphosphate, such as alendronate (ALN), is widely used in osteoporosis treatment to target macrophages such as osteoclasts.^130,131^ Upon uptake, ALN forms an ATP analog in macrophages, interfering with ATP homeostasis, and this may indirectly influence endosomal v-ATPase and H^+^ concentrations. Furthermore, the glucocorticoid, dexamethasone (Dex), has already been used to inhibit pathological inflammation in SARS-CoV-2-infected patients.^132,133^ Given the influence on the phenotype of AMs, Dex might also regulate endosomal pH or other factors. In our experiments, we found that Dex downregulates CTSL expression in AMs and that ALN increases endosomal pH. Therefore, although conventional systemic administration of Dex and ALN faces the challenge of alveolar surfactant, which prevents the drug molecules from gaining access to AMs, airway delivery of these two drugs is able to directly target AMs by complementarily interfering with the endosomal release of SARS-CoV-2 RNA by through altered CTSL expression and endosomal pH, respectively.^134^ Dex is a well-known anti-inflammatory drug, that inhibits AMs from releasing proinflammatory cytokines, reducing AMs-induced lung pathological damage. In addition, Dex has been shown to polarize macrophages toward the M2 phenotype,^135^ and higher CTSL expression in M1 macrophages than in M2 macrophages has been observed.^134^ Overall, upon airway administration, Dex and ALN locally regulate endosomes of AMs and prevent release of SARS-CoV-2 RNA from endosomes, thus achieving the goal of preventing or controlling viral infection at a very early stage (Fig. 4b). In addition, such medication is easy, safe, low-cost, and broad-spectrum in the management of SARS-CoV-2 variant infection.

### Challenges and future perspectives

In the first two decades of the 21st century, two types of coronaviruses have swept across the world, especially SARS-CoV-2, which is still spreading globally at present. Vaccines and neutralizing antibodies have been shown to be effective in the control of viral spreading, which, unfortunately, are challenged by the high frequency of viral mutations.^136–139^ The Current Omicron variant has evolved outrageous transmissibility, which harbors up to 36 mutations in the S protein but generates milder symptoms, increased frequent asymptomatic carriers and decreased hospitalization.^140–142^ Despite the reduced virulence, people are continually afraid of the generation of the new mutated virus from Omicron, which has an increased virulence and can bring the next wave of catastrophe. This worry is not a groundless concern, considering the aspects, including (1) strategies and policies on the control of viral spread are different among countries with different political systems; (2) this world is a global village with convenient and rapid transport systems and large interconnected populations and rapid; (3) the ecosystem is losing balance due to immoderate human activities; and (4) SARS-CoV-2 can infect a variety of animals. Facing these challenges, current advances in science and technology lag behind the requirement for prevention and treatment of SARS-CoV-2 infection as well as other new infectious diseases. However, this situation might be reversed by currently strengthened financial support from governments, funds and enterprises. The development of a new generation of vaccines is of paramount importance. Antiviral medications are also important as an adjunct to vaccines in the control of viral infection. Apart from these strategies, the alveolar macrophage-based strategy is worthy of paying attention to, considering the unique anatomic structure of alveoli. As the first-line defenders, AMs effectively take up viruses and destroy them without induction of inflammation under certain conditions. This is exactly the outcome we want, the clearance of the viral infection without immunopathology.

## Concluding remarks

Lethal respiratory viruses mainly include avian flu (H5N1 and H7N9), MERS-CoV, SARS-CoV, and SARS-CoV-2.^143^ Studies have shown that these viruses could infect AMs in the lower respiratory tract, causing fatal pneumonia.^143^ In particular, MERS-CoV not only infects but also effectively replicates in human monocyte-derived macrophages (MDMs), leading to extensive cytotoxicity and a high level of proinflammatory cytokines.^144^ The alveoli are the terminal end of the lower respiratory tract, with the PS on the surface. Therefore, in steady-state, PS might protect ATII cells from infection. However, when viruses replicate in AMs, excessive proinflammatory cytokines are produced, impeding the generation of PS. Without the insulation provided by PS, viruses can directly enter ATII cells. Moreover, the type I interferon released by infected M1 AMs directly stimulates ATII cells to produce mucus through the AhR-dependent pathway, which may deposit on the surface of alveoli.^103^ Thus, the damage to the alveolar structure and the formation of a mucous layer on alveoli lead to impaired O_2_ and CO_2_ exchange (Fig. 1). In conclusion, viruses invading the lower respiratory tract may be fatal because they cause breathing difficulty. Recently, several studies have shown that Omicron primarily invades bronchus epithelial cells and spreads faster than in alveoli,^145,146^ which may explain why Omicron variant-infected patients have less severe symptoms than Delta-infected patients. In these processes, macrophages are the common mechanism. We speculate that ALN and Dex may be applied in the prevention and treatment of other lethal respiratory virus infections.
